# Learning different task spaces: how explored density aligns the Quiet Eye

**DOI:** 10.1007/s10339-022-01090-5

**Published:** 2022-05-09

**Authors:** André Klostermann, Florian Reinbold, Ralf Kredel

**Affiliations:** Institute of Sport Science, University of Bern, Bremgartenstrasse 145, CH-3012 Bern, Switzerland

**Keywords:** Motor learning, Perception–action, Inhibition, Task space

## Abstract

In the current study, predictions of a theoretical account to the explanation of the Quiet Eye (QE) were investigated. To this end, by manipulating the learning environment, participants (*n* = 52) learned an underhand throwing task which required to explore task-solution spaces of low vs. high density over a 4-week training phase (640 training trials). Although throwing performance was improved, surprisingly, in posttest and retention test shorter QE durations were found. It is speculated that on a short-time learning scale this effect might be explained by more efficient information processing. Moreover, a trend was observed which suggests that—in line with the inhibition hypothesis—when exploring high-density task-solution spaces longer QE durations are required. However, the rather small effect sizes necessitate further research, which will allow to manipulate the response–effect mappings more directly as, for example, in virtual environments.

## Introduction

When performing far-aiming tasks, athletes show a distinct quietness in their eye movements when performing their movements. For example, in basketball free-throw players maintain visual focus at the hoop before and while shot execution (e.g., Harle and Vickers 2001). However, this behavior can not only be found in basketball but in many different sports, like golf (e.g., Vine et al. 2011), rifle shooting (e.g., Mann et al. 2011), darts (e.g., Vickers et al. 2000), or billiard (e.g., Williams et al. 2002). Until today, this phenomenon has been described in more than 25 different motor tasks; some of them also outside of the sports domain (Vickers 2016).

Vickers (1996) was the first to reveal this phenomenon in Olympic basketball players, which she then termed the Quiet Eye (QE) and which denotes an optimal coupling between visual perception and motor action. It allows crucial visual information to be available when it is required the most, i.e., just before the critical part of the task is being executed. Meanwhile, the QE is defined as the last visual fixation at a specific object or location in space before the onset of the critical movement phase. For example, in basketball free throw the QE starts when the athlete visually fixates the rim of the hoop before starting the final extension of the throwing arm (i.e., just before ball release). The QE has its offset when the fixation is dissolved (definition adapted from Vickers 2007). Meanwhile, there is strong empirical evidence, supported by several meta-analyses and reviews (Gonzalez et al. 2017a, b; Klostermann and Moeinirad 2020; Lebeau et al. 2016a, b; Mann et al. 2007; Rienhoff et al. 2016), which identified the QE as a characteristic of motor expertise and motor performance. In the classic study by Vickers (1996), it was found that better free-throw shooters—all participants being members of the Olympic national team—showed nearly double the QE duration as compared to the worse free-throw shooters. Moreover, the better free-throw shooters showed again about 20% longer QE durations in successful as compared to unsuccessful free-throw attempts. Both results—longer QE durations in high-level athletes and in successful motor performance—were often replicated, also in more controlled experimental studies. In terms of motor learning, in a wealth of studies Vine, Wilson, and colleagues (for an overview, Vine et al. 2014) demonstrated the potential of QE interventions in skill training, among others showing accelerated motor learning in QE-trained basketball players and golf players. Among others, Klostermann et al. (2013) revealed a close relation of long QE duration to motor performance. Under experimentally controlled long vs. short QE-duration conditions, participants showed improved throwing performance when having long QE durations (see also, e.g., Sun et al. 2016; but see also Harris et al. 2021).

Derived from a cognitive perspective, Vickers (1996) suggested that during the QE duration visual information is fed into the sensorimotor system enabling precise fine-tuning of the movement parameters (Vickers 1996). Williams et al. (2002) provided empirical support for this hypothetical link between the QE and movement parametrization as they revealed strong positive relations between task demands (interpreted as prolonged periods of cognitive response programming) and the QE duration. Meanwhile, further empirical evidence comes from studies applying observational designs as the study by Mann et al. (2011) who studied neurophysiological correlates over the QE duration as well as from studies applying experimental designs. As one representative of the latter category, Gonzalez et al. (2017a) investigated the QE of elite and novice archers in a virtual archery task under high-noise vs. low-noise conditions (i.e., visual perturbations of motor performance). By means of a joystick, the participants aimed a crosshair at a virtual target disk and released the virtual arrow by pressing a response button. In the high-noise condition, the crosshair’s path was manipulated by adding random noise and, thus, required constant online corrections prior to release. As predicted, both groups showed inferior performance in the high-noise condition, thus confirming the manipulation. However, only the elite archers showed longer QE durations in the high-noise as compared to the low-noise conditions. This raises the question whether movement parametrization itself explains the advantage of long QE durations as increased information processing should be reflected in QE durations independent of the expertise level (e.g., Williams et al. 2002).

Already Mann et al. (2016) questioned the explanatory power of the movement-parametrization hypothesis (cf. Vickers 1996), stating that it remains unclear why highly skilled athletes require longer QE duration if efficiency is paramount. Likewise, from an information-processing approach it seems elusive to expect higher processing demands in skilled athletes who otherwise excel by highly automatized processes (e.g., Ericsson et al. 2018). Thus, a reduction of the functionality of the QE to augment the fine-tuning of movement parameters might not tell the whole story. Consequently, Gonzalez et al. (2017a) speculated that the QE might serve as an inhibitory function which allows the elite archers to maintain visual attention by blocking competing cognitive resources. The idea of an optimal attentional control already was put forward by Vine, Wilson and colleagues (e.g., Vine and Wilson 2010) as they found QE-trained athletes to maintain performance in a golf-putting task under raised pressure conditions and, thus, to maintain attentional control. However, specific predictions regarding this efficiency paradox were not tested yet.

An alternative approach was put forward by Klostermann et al. (2014) who also assumed an inhibitory function to explain the QE effect. However, instead of relating this inhibition to attentional control mechanisms, it was assumed that the QE subserves the shielding of the movement parameterization for the currently selected from alternative, potentially viable task variants and parameterizations. Neumann (1987; see also Allport 1987) theorized that the shielding over response selection and programming is necessary “to avoid the behavioural chaos that would result from an attempt to simultaneously perform all possible actions for which sufficient causes exist” (p. 374). Empirical evidence regarding this inhibitory mechanism stems from a wealth of studies investigating the effects of distractors, introduced as optional targets in manual reach-to-grasp movements. Generally, it is found that including optional targets affects hand trajectories (e.g., Howard and Tippert 1997) unless this alternative response selection can be successfully inhibited (e.g., Welsh and Elliot 2004; see also Cisek and Kalaska 2005).

The proposed inhibition function of the QE in response selection was shown, among others, in ambiguous response-selection conditions in far-aiming tasks by Klostermann (2018, 2020). In these studies, participants threw balls as precise as possible on virtual targets presented at a life-sized screen. Response-selection demands were manipulated by having participants select either one target out of one target and out of two targets vs. one target out of four targets. In all three studies, longer QE durations were found if participants had to select one target out of four targets, in particular if the targets were grouped closely together, thus, shared similar three-dimensional positions and, in turn, required similar response selection. Consequently, increased inhibition demands indeed seem to prolong the QE duration required for successful action specification.

Transferred to the QE expertise effect (i.e., longer QE duration in skilled athletes), Klostermann et al. (2014) suggested that skilled athletes require long QE durations because they must shield one action specification against potential viable action specifications accumulated over years of skill training. When visualized as task-space landscapes (Hossner et al. 2020), skilled athletes would have developed a very dense task-solution space for those specifications which entail a high probability of task-goal accomplishment. In contrast, less-skilled athletes encounter lower shielding demands because of the comparably low number of potentially viable and rather dissimilar action specifications (i.e., they have developed a task-solution space of low density), thus requiring shorter QE durations. The suggested density in task-solution spaces can be exemplified by means of the especial skill effect (cf. Keetch et al. 2005) which occurs “when performance of a single action from within a class of actions produces an advantage in performance”. For example, in basketball set shots, it was shown that the set shot from the distance of the free-throw line entails this performance advantage as compared to other set shots performed from other distances due to the large amount of associated practice. Consequently, one should suggest similar effects for the QE duration, which is exactly what was found by Klostermann (2019). Similarly, Flindall et al. (2020) recently showed that the otherwise linear relation between task demands and the QE duration (e.g., Walters-Symons et al. 2018; Williams et al. 2002) is broken if dart experts throw darts from longer distances or with the non-dominant hand. Thus, there is some empirical evidence from performance studies to support the inhibition hypothesis (see also, Gonzalez et al. 2017b).

Based on the empirical findings sketched above and the results of a previous learning study (Klostermann and Hossner 2018), the current experiment further investigated the hypothesized relation between the QE duration, motor expertise, and task-space density. To this end, participants learned an underhand throwing task which required to throw balls with their non-dominant hand as centrally as possible on projected target disks. The participants trained with two different learning regimes. The high-density group practiced at target disks that were positioned in increasingly smaller vertical distance to the test target. In contrast, the low-density group trained with target disks that were increasingly positioned further away from the test target. Thus, with different time lags to the posttest and the retention test, the groups experienced rather similar (increasing density) vs. dissimilar (decreasing density) action specifications in the training and test trials, respectively. Consequently, a larger increase in QE duration from pretest to posttest and retention test for the high-density vs. the low-density group was predicted.

## Methods

### Participants

With the expectation of a medium effect size (*f* = 0.25), power set to 1 − *β* = 0.95 and an expected correlation among repeated measures of *r* = 0.4, a priori sample size estimation revealed—for the predicted interaction effect—an optimal sample size of *n* = 52 (calculated with G*Power 3.1; cf. Faul et al. 2009). However, to compensate for potential dropouts a total of fifty-seven sport-science students were drawn. Five participants had to be excluded from further data analyses because of missing test and or training data. The remaining 25 male (age: M = 21.9 years, SD = 3.3 years) and 27 female (age: M = 20.4 years, SD = 1.2 years) participants had all self-reported normal vision or corrected-to-normal vision by wearing lenses and were right-handed. Balanced by the pretest throwing performance (i.e., radial error, in mm) and the QE data (i.e., QE duration, in ms), the participants were assigned into a high-density vs. low-density training group. All participants received course credits in return and were unaware of the research question. The protocol was approved by the ethics committee of the local Faculty of Human Sciences and was carried out in accordance with the 1964 Declaration of Helsinki.

### Apparatus

Participants stood in front of a white screen (width: 3.2 m, height: 2.2 m), on which the target disks (diameter: 125 mm) were projected by a LCD projector (Epson H271B LCD Projector, Nagano, Japan). Ball, head, and hand movement trajectories were collected with a 3D motion-capture system (VICON T20, VICON Motion Systems Limited, Oxford, United Kingdom, 200 Hz). The eye tracker (EyeSeeCam, EyeSeeTec GmbH, Fürstenfeldbruck, Germany, 220 Hz) was integrated in the VICON system and connected via an active optical FireWire extension (GOF-Repeater 800, Unibrain, San Ramon, CA, USA) to a MacBook Pro (Apple, Cupertino, CA, USA), on which the EyeSeeCam software was used for calibrating the eye tracker and streaming eye orientation data over the network. The internal loudspeaker of the main experimental control workstation (HP Z230 Tower-Workstation, Hewlett Packard, Palo Alto, CA, USA) played the audio signals, which were used to structure the timing of the experiments.

Throwing movement and ball flight were assessed by passive retro-reflective markers mounted to a marker cluster (marker diameter: 14 mm) and retro-reflective balls (ball diameter: 50 mm), respectively. The marker cluster was attached to a fingerless glove on the throwing hand by use of velcro tape. The EyeSeeCam assessed the orientation of the left eye by means of an optical tracking of the corneal reflections from infra-red light. The eye orientation data were streamed in real time via Ethernet to the control workstation, which additionally received synchronized positional and rotational head movement data via the retro-reflective markers attached to the EyeSeeCam. With these data, a custom MATLAB (Matlab 2014a, The MathWorks, Natick, MA, USA) software application calculated the three-dimensional gaze vector in the laboratory frame of reference. The accuracy of this integrated eye-tracking system amounts to 0.5° of visual angle with a resolution of 0.01° RMS within 25° of the participant’s field of view (cf. Kredel et al. 2015).

The visual stimuli were programmed in MATLAB 2016b (The Mathworks Natick, MA, USA), and the resulting AVI video files were rendered with Magix Video Pro X3 (Magix Software GmbH, Berlin, Germany) into a MP4 container format with an H.264 compression (resolution: 1280 × 960 px). Data analyses were conducted with MATLAB 2016b, Microsoft Excel 2016 (Microsoft, Redmond, WA, USA), and IBM SPSS Statistics 27 (IBM, Armonk, NY, USA).

### Visual stimuli

**Fig. 1:**
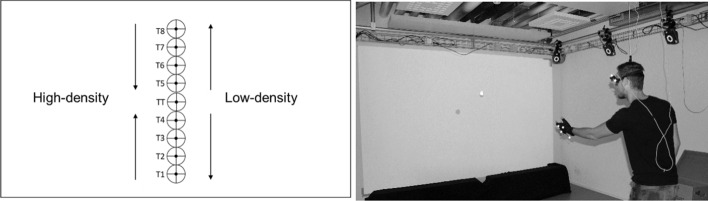
Illustrated positioning (left) of the training (T1–T8) and test (TT) targets at the screen including a visualization of the two different intervention regimes (high-density vs. low-density) and a picture (right) showing the experimental laboratory setup as well as the throwing task

At the beginning of each trial, either after 1000 ms or after 1300 ms a fixation point was presented either 960 mm off-center to the left or 960 mm off-center to the right with respect to the central point of the screen. After the presentation of an auditory start signal (randomly played between 2200 and 2500 ms after trial start), the target disk was presented horizontally centered at eight different heights, i.e., (from bottom until top) 300 mm (T1), 500 mm (T2), 700 mm (T3), 900 mm (T4), 1300 mm (T5), 1500 mm (T6), 1700 mm (T7), and 1900 mm (T8). Consequently, after the presentation of the fixation point, participants had to saccade to each of the 9 potential target positions. In the test trials, the target always was shown at a height of 1100 mm (TT) (see Fig. 1, left). Each trial lasted 10000 ms with the target disk disappearing after 6000 ms.

### Procedure

The experiment was conducted in the institute’s sensorimotor laboratory in individual sessions. After the pretest, participants conducted four learning sessions which were separated by (roughly) seven days. The posttest was conducted directly at the end of the last learning session, and the retention test was delayed by one week.

At the first day, participants received brief instructions and provided informed consent. Subsequently, participants were equipped with the VICON marker cluster glove as well as the EyeSeeCam and were shown an introductory video. The EyeSeeCam was then calibrated, which required participants to consecutively fixate five equidistant points (at 8.5° of visual angle) on the life-sized screen. The EyeSeeCam was re-calibrated if the point of gaze deviated by more than 1° of visual angle from one of the points of the calibration grid, which was checked after every eighth test trial. In each trial, one ball had to be picked out of the ball box positioned at hip height next to the participants. Participants were then asked to fixate the fixation cross until the presentation of the auditory start signal. After the appearance of the target disk, the ball had to be thrown as centrally as possible at the respective target disk with the non-dominant hand using an underhand throwing technique. The participants were centrally positioned in front of the screen at a distance of about 3200 mm (see Fig. 1, right).

On the testing days (pretest, posttest, and retention test), the participants were tested in 2 blocks of 16 trials each at the target TT. The pretest and the retention test were preceded by a 10-trial warm-up block. At the learning days (L1 until L4), participants trained in 10 blocks of 16 trials each. In the high-density group, participants trained predominantly at L1 with the targets T1 and T8 (37.5% of all training trials, each), at L2 with the targets T2 and T7 (37.5% of all training trials, each), at L3 with the targets T3 and T6 (37.5% of all training trials, each), and at L4 with the targets L4 and L5 (37.5% of all training trials, each). At each learning day, the remaining target positions (e.g., targets T2–T7 at L1) were evenly distributed among the remaining training trials. In the low-density group, participants trained the reversed order, thus, at L1 with predominantly T4 and T5 and at L4 with predominantly T1 and T8. Thus, whereas the high-density group experienced increasingly similar action specification, the low-density group experienced increasingly dissimilar action specifications in regard to the target TT. On average, 6.4 days (SD = 1.0 days) elapsed between the pretest and L1, 6.7 days (SD = 0.7 days) between L1 and L2, 7.3 days (SD = 0.9 days) between L2 and L3, exact 7 days between L3 and L4/posttest, and 7.4 days (SD = 0.9 days) between L4/posttest and the retention test. The testing sessions lasted about 30 min and the learning sessions about one hour. At the end of the retention test, the participants were thanked and informed about the aims of the study.

### Measures

In pretest, posttest, and retention test, all trials without a valid QE detection and with technical issues (i.e., invalid detection of the ball flight) were removed from further data analyses. Thus, in the pretest on average 17.4% of all trials (SD = 13.3%), in the posttest on average 18.1% of all trials (SD = 11.1%), and in the retention test 16.8% of all trials (SD = 10.4%) could not be analyzed. This means that out of 32 test trials, in the pretest on average 26.4 trials, in the posttest, 26.2 trials, and in the retention test 26.6 trials were used to calculate the dependent measures. Differences in missing trials between training groups were small (pretest: *N* = 1.0 trials; posttest: *N* = 0.7 trials, retention test: *N* = 0.2 trials). The analyses and the results of the training data are reported in Appendix 1.

#### Quiet Eye

The gaze data were analyzed using the dispersion-based algorithm by Nyström and Holmqvist (2009), which classifies a fixation as soon as the point of gaze becomes stable within a circular area of 1.2° of visual angle for at least 120 ms (for more details, see Kredel et al. 2015). The QE was defined as the final fixation on the target disk before the initiation of the hand’s forward swing. The onset and offset were identified as the first and last VICON frames of the QE fixation, respectively. QE onset and offset were then calculated as relative values in relation to the initiation of the forward swing. Thus, negative values represent moments in time before the initiation of the forward swing, whereas positive values represent moments in time after the initiation of the forward swing. The QE duration was calculated as time interval between QE onset and QE offset. The initiation of the forward swing was determined as the next VICON frame after the average position of the hand reached its local minimum in the sagittal plane before ball release (i.e., one VICON frame after the hand reaching its backmost position; see also Klostermann et al. 2013). QE onset, offset, and duration were separately aggregated for the 3 (test: pretest, posttest,  and retention test) times 2 (training group: high-density vs. low-density) factors. Moreover, median splits of QE duration were performed to assess effects of short vs. long QE durations on throwing performance (cf., e.g., Causer et al. 2017; Klostermann 2018).

#### Throwing performance

Throwing performance was obtained by computing radial-error scores. To this end, the position of the center of the target disk was determined by converting the relative position of the target in the video scene to the physical screen’s frame of reference. The metric deviation of the ball from the target center at ball impact could then be calculated. The throwing performance was separately aggregated for the 3 (test: pretest, posttest, and retention test) times 2 (training group: high-density vs. low-density) factors as well as for long vs. short QE-duration trials.

### Statistical analyses

QE duration, QE onset, and QE offset were analyzed with mixed-factorial 3 (test: pretest, posttest, and retention test) times 2 (training group: high-density vs. low-density) ANOVAs with repeated measures on the first factor. In addition, throwing performance was analyzed with mixed-factorial 3 (test: pretest, posttest, and retention test) times 2 (training group: high-density vs. low-density) times 2 (split: long QE duration vs. short QE duration) ANOVAs with repeated measures on the first and the last factors (e.g., Vickers 2016). A posteriori effect sizes were computed as Cohen’s *d* values and partial eta squared, *η*_p_^2^. In case of violations of the sphericity assumption, Greenhouse–Geisser corrections were applied.

## Results

### Quiet Eye

**Fig. 2:**
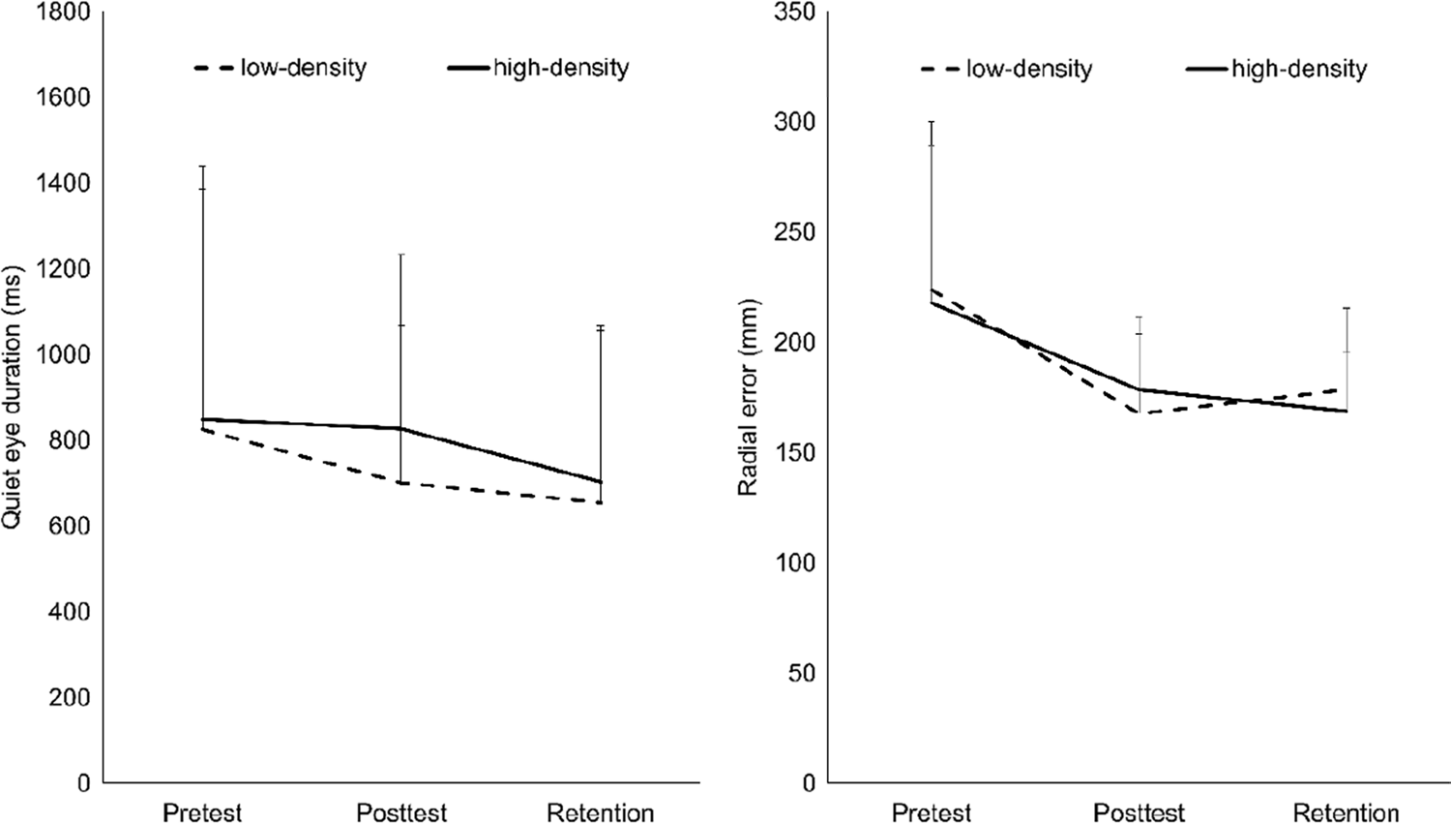
Average Quiet Eye duration (left, ms, SD) and radial error (right, mm, SD) as a function of test (pretest, posttest, and retention test) and training group (low-density vs. high-density)

For QE duration (see Fig. 2, left), a significant main effect for test was found, *F*(1.7, 83.4) = 3.59, *p* < 0.05, *η*_p_^2^ = 0.07. Other than predicted, participants showed significantly shorter QE durations at retention test when compared to pretest, *t*(50) = 2.56, *p* < 0.05, *d* = 0.36. The predicted interaction effect, *F*(1.7, 83.4) = 0.40, *p* > 0.05, *η*_p_^2^ = 0.01, and the main effect for training group (*p* > 0.05, *η*_p_^2^ = 0.01) were not significant. For QE onset and QE offset (see Table 1), no significant main effects (all *p*s > 0.10, all *η*_p_^2^ < 0.05) and interaction effects (all *p*s > 0.23, all *η*_p_^2^ < 0.03) were revealed.

**Table 1:** Average (± *SD*) QE onset, QE offset, and radial error for long (RE-long) vs. short (RE-short) for the low-density vs. the high-density group as a function of test day (pretest, posttest, and retention test)

		Low-density	High-density
Pretest	QE onset	− 429.8 ms (± 186.8 ms)	− 518.3 ms (± 251.4 ms)
	QE offset	390.5 ms (± 512.7 ms)	326.1 ms (± 469.8 ms)
	RE-long	213.5 mm (± 80.8 mm)	206.1 mm (± 72.3 mm)
	RE-short	234.2 mm (± 76.9 mm)	227.6 mm (± 78.1 mm)
Posttest	QE onset	− 428.9 ms (± 190.8 ms)	− 469.1 ms (± 182.8 ms)
	QE offset	267.1 ms (± 333.6 ms)	352.8 ms (± 257.6 ms)
	RE-long	166.2 mm (± 41.5 mm)	183.6 mm (± 41.8 mm)
	RE-short	168.6 mm (± 37.2 mm)	173.3 mm (± 34.8 mm)
Retention test	QE onset	− 416.9 ms (± 157.5 ms)	− 408.6 ms (± 177.6 ms)
	QE offset	232.5 ms (± 320.5 ms)	289.6 ms (± 324.8 ms)
	RE-long	174.5 mm (± 35.1 mm)	169.6 mm (± 36.9 mm)
	RE-short	181.6 mm (± 47.6 mm)	167.9 mm (± 33.8 mm)

### Throwing performance

As can be seen in Fig. 2 right, a significant main effect for test, *F*(1.29, 63.3) = 17.93, *p* < 0.05, *η*_p_^2^ = 0.27, was found with smaller radial errors in posttest and retention tests as compared to the pretest (all *p*s < 0.01, all *d*s > 0.61). The further differentiation for long vs. short QE-duration trials (see Table 1) revealed a significant test times split interaction, *F*(2, 98) = 4.97, *p* < 0.05, *η*_p_^2^ = 0.09. Only at pretest (*p* < 0.01, *d* = 0.45), significantly lower radial errors in long vs. short QE-duration trials were found. The overall performance advantage of long vs. short QE-duration trials was rather small and not significant, *F*(1, 49) = 3.28, *p* > 0.05, *η*_p_^2^ = 0.06.

## Discussion

In this experiment, participants learned a far-aiming task with two different interventions to empirically test the suggested relation between the explored density of the task-solution space and the development of the QE duration. To this end, participants either trained with a high-density protocol with constantly decreasing training-to-test-target distances or with a low-density protocol with constantly increasing training-to-test-target distances. Based on the assumption that a dense task-solution space requires long QE durations, it was predicted that particularly the high-density training group would show increased QE duration at posttest and retention test.

The data revealed that both groups—starting with similar throwing performance and QE durations—improved their throwing performance due to training but showed *reduced* QE durations at posttest and retention test (see Fig. 2). This surprising result is not only in stark contrast to earlier studies with specific QE-training regimes (e.g., Vine and Wilson 2010, 2011) but also to earlier motor learning studies (e.g., Klostermann and Hossner 2018). Thus, at first sight this result seems puzzling. But it might tie in with the suggested economization of information processing (Mann et al. 2016), which assumes that with increasing motor experience movement parametrization requires less cognitive resources (e.g., Ericsson et al. 2018; see also Fitts and Posner 1967) and, thus, should also require shorter QE durations. Consequently, in pretest, participants controlled their throwing movement rather consciously step by step (Beilock and Carr 2001) as they had to attain several sub-goals before reaching the final goal (e.g., Greenwald 1970). This, in turn, required high amount of information processing and control effort, respectively, (e.g., Weaver 2015) which, in turn, required long QE durations. With increasing motor experience, the control effort decreased since the throwing task became less complex due to several sub-goals being chained to longer movement sequences (Greenwald 1970). Consequently, motor control became more automated and efficient which required less information processing and shorter QE durations. But how does this explanation fit into the QE literature? First, available evidence of reduced QE durations with increasing motor expertise is scarce (for an overview, e.g., Lebeau et al. 2016a, b). Nevertheless, it might, at least theoretically, be plausible that over motor learning, QE duration does not increase linearly but instead shows a U-shaped development. Since most QE expertise studies investigated only differences between less skilled and highly skilled athletes, this development might have been overseen so far. Thus, the (implicit) assumption of a constant increase might simply be wrong. Instead, the initial increase in efficiency in motor control and the respective reduction in information processing might indeed result in a drop in QE duration in a first phase. Only after a considerable amount of training when athletes achieve high stability in motor performance, the number of potential and more similar action specifications steadily increases requiring longer QE durations to shield a selected against alternative parameter specifications. However, this interpretation remains very speculative and requires further studies that apply (1) more elaborated biomechanical models to further specify the suggested learning phases, (2) specific research methods to measure cognitive load (e.g., Carnegie et al. 2020) and (3) longer learning phases to more closely and for longer durations track the development of the QE over motor learning.

The current data further suggest that the QE development differed between the two training groups. However, we acknowledge that the predicted interaction effect for QE duration clearly *failed* to reach significance. Nonetheless, the trends observed between the two groups merit discussion as they might help to gain further insights into the underlying mechanisms. Thus, when returning once more to Fig. 2 (left), it can be seen that the high-density group rather maintained the QE duration from pretest to posttest (*d* =  − 0.04). In contrast, the low-density group showed an immediate drop from pretest to posttest (*d* = − 0.24). These differences at posttest reached a small effect size of *d* = 0.31. At the retention test, both groups showed further drops in QE duration, which already was observed in earlier QE learning studies (Klostermann and Hossner 2018).

Accordingly, the descriptive analyses and the respective effect sizes at least do not exclude that learning a dense task-solution space requires longer QE durations. Obviously, with the previous experimental designs (see also Klostermann and Hossner 2018) it was not possible to empirically test our hypothesis. Therefore, to follow-up on the results of this experiment one could increase sample size, thus increasing statistical power to inference-statistically harden this small effect. This, however, when expecting a similar interaction effect (*η*_p_^2^ = 0.01) and accepting a power of 1−*β* = 0.80, requires a sample size of at least *n* = 162 participants questioning the feasibility of the study.

Therefore, an alternative approach is favored which necessitates to further push the effect in motor learning that—to be kept in one’s mind—typically can only be found with large amount of training hours (i.e., in highly skilled athletes). Thus, instead of training the learners into task-solution spaces with different densities by manipulating constraints of the learning environment, a more direct approach is required that allows—by using a virtual-reality setup—to manipulate movement-effect mappings over the learning phase more directly. As virtual environments (VEs) allow a user-defined specification and manipulation of the applied physics, for example, ball-flight trajectories could be manipulated in such a way that over the learning phase participants experience experimentally controlled high-density vs. low-density task-solution spaces. Exactly such an experimental paradigm is currently being developed in our laboratory and will be applied soon.

A limitation of the current research—obviously—resides in the comparably small effects that were revealed. But once again, one should have in mind that our research aims to uncover mechanisms that in real life develop throughout several years of motor practice. Thus, although the studies had a fair amount of training trials (more than 600 trials) and a comparably long training duration (longer than 3 weeks of training), this still does not equate to the amount of experience in highly skilled athletes. Consequently, it is to the researchers to develop experimental paradigms which allow to reveal the underlying mechanisms within feasible time frames and extents of research projects. Thus, when reflecting on the experience gathered, the future in this field of research, indeed, might reside in the application of VEs, which, among others, allow to manipulate physical laws. Whether or not the current results and—more generally—to what extent findings from VR studies can be transferred into the real-world situation still needs to be answered. However, first empirical studies at least suggest that athletes behave similar when performing in VR and in the respective real-world setting as long as the crucial environmental and task properties and interaction possibilities are sufficiently similar (e.g., Michalski et al. 2019). Nonetheless, with all the advantages that come along with the application of VR settings, researchers always should be aware of potential limitations in the external validity of their research.

## Figures and Tables

**Fig. 3:**
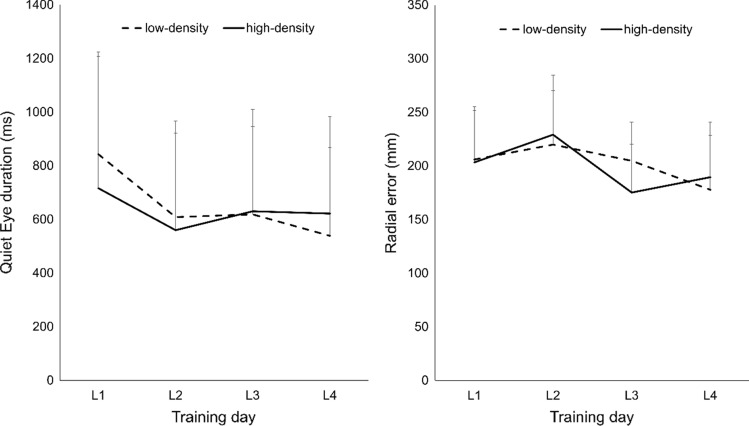
Average Quiet eye duration (left, ms, SD) and radial error (right, mm, SD) as a function of training day (L1–L4) and training group (low-density vs. high-density)

## Data Availability

After publication, all relevant data will be made available in the [Name of the repository].

## Training data: Quiet Eye and throwing performance

To track the learning progress, the last two training blocks at the training days L1–L4 were recorded and analyzed (i.e., two blocks of 16 trials, each). Due to technical issues, however, the data sets of 9 participants at L1, of 8 participants at L2 and of 2 participants at L3 could not be analyzed. To avoid bias, only participants with full data sets over the learning were considered for subsequent data aggregation. Because of the severe data loss, no inferential statistics were performed on the training data.

Results showed that the two groups started with slightly different QE durations at L1 with longer QE durations on the low-density as compared to the high-density group (see Fig. 3, left). Whereas, in the following, the low-density group continuously decreased the QE from L1 until L4, the high-density group, although some variation from training day until training day was evident, rather maintained the QE duration. At L4 the high-density group had an average QE duration of 621.7 ms (SD = 360.7 ms) and the low-density group had an average QE duration of 538.7 ms (SD = 329.9 ms). Thus, compared to L1, the high-density group decreased their QE duration about 36% and the low-density group showed a minor decrease of 13% only. With regard to the performance data (Fig. 3, right), after a small increase in radial error from L1 to L2, both groups showed a continuous decrease in the radial error until L4. At L4, both groups had a radial error of about 182 mm, thus, 11.5% smaller radial errors as compared to L1.
